# Tularaemia after tick exposure - typical presentation of rare disease misdiagnosed as atypical presentation of common diseases: a case report

**DOI:** 10.4076/1757-1626-2-7954

**Published:** 2009-07-31

**Authors:** Karolina Switaj, Maria Olszynska-Krowicka, Hanna Zarnowska-Prymek, Piotr Zaborowski

**Affiliations:** Department of Zoonotic and Tropical Diseases, Medical University of Warsawul. Wolska 37, 01-201 WarsawPoland

## Abstract

A 44-year-old female was admitted because of tender, enlarged inguinal lymph nodes with a history of tick bite five weeks earlier. In the place of a tick bite on the skin a small ulcer was present. The primary symptoms before admission suggested typical diseases related to tick bite such as Lyme borreliosis and tick-borne encephalitis, what corresponded with positive IgM ELISA test for Lyme borreliosis. The course of disease however clarified the diagnosis of tularaemia, which is a relatively rare disease in Poland (6 cases per 40 million population are reported annually). The ultimate diagnosis was confirmed by serological tests.

## Introduction

Tularaemia is zoonosis caused by gram-negative, aerobic coccobacillus *Francisella tularensis* which has in recent years been considered a possible biological weapon agent [1-3]. At present, there are four subspecies recognized: *tularensis, holarctica, mediasiatica*, and *novicida* [4]. Tularaemia occurs primarily in the Northern Hemisphere from approximately 30° to 70° north latitude. Hares, rabbits and rodents are the main animal reservoirs of the pathogen. Biting arthropods such as ticks and deerflies are important vectors. Mosquitoes have also been identified as vectors [5]. The subspecies *novicida* is related to water-borne transmission. Direct contact with infected animals or animals’ tissues can also result in tularaemia infection either.

The clinical presentation depends among others on virulence of *F. tularensis* subspecies and the route of pathogen invasion to the host organism (through the skin or mucous membranes). Subspecies *tularensis* isolated only in North America is the most virulent one. The course of pneumonic or typhoidal tularaemia can be severe.

## Case presentation

A 44-year-old Caucasian female (164 cm, 68 kg), farmer was admitted because of tender mass in her left groin and numbness in her hands and feet after a tick bite in the left lumbar area five weeks before admission. The patient was a non-smoker, four times pregnant, negating chronic diseases except for hypertension reduced by 2.5 mg of ramipril.

Soon after removal of the tick she was seen by general practitioner who administered oral amoxicillin because of skin redness in the place of the tick bite. Two weeks later she developed high fever (39°C) and was seen in the outpatient clinic of Warsaw Hospital for Infectious Diseases where the treatment was changed into oral doxycycline (2 × 100 mg) and in suspicion of the first phase of tick bite encephalitis the patient was advised that in the case of headache or worsening of general condition she should be seen in the consulting point of Warsaw Hospital for Infectious Diseases. Ten days later she came to the consulting point in better general condition however suffering a severe pain in her left groin accompanied by the feeling of numbness in her hands and feet. She was admitted to the hospital to the Department of Zoonotic and Tropical Diseases. On admission: the body temperature was normal, neck stiffness was absent, contact with the patient was normal. On the skin of the left lumbar area, in the place of the tick-bite, an ulcer of about 1.5 cm in diameter was present. In the left groin the tender, swollen lymph nodes were palpable. No other abnormalities on physical examination were present. Accessory investigations such as blood count and smear were within normal limits. CRP was not elevated. Urinalysis showed changes characteristic for infection- at urinary microscopy 50-70 white blood cells/hpf. The serology of Lyme borreliosis (ELISA) was positive for IgM and a sample for serology of tularaemia was processed.

The ultrasound scan of the abdomen and the groins revealed no abnormalities except for hypoechogenic area - the complex of inflamed lymph nodes 18 × 12 × 20 mm- and a single lymph node 20 mm in diameter in the left inguinal region.

Oral doxycycline was changed into intravenous (2 × 100 mg), penicillin G (4 × 5 mln i. U.) and metronidazole (3 × 500 i.v.) were added.

Significant improvement in the left inguinal region was observed in about a week. The result of agglutination test with antigens of *F. tularensis* was available and indicated positive reaction in high dilution. The i.v. therapy with antibiotics was continued for 3 weeks followed by one week of oral doxycycline (2 × 100 mg).

During control examination one month after therapy termination the patient reported complete recovery and the skin ulcer was healed. The ultrasound scan of the left inguinal region showed solitary lymph nodes 11-12 mm, without necrosis.

The western blotting test of IgM for Lyme borreliosis from the sample taken on the admission to the hospital (at the beginning of the disease) was available at that time. It was negative indicating that the result of ELISA test for Lyme borreliosis was false positive.

## Discussion

Tularaemia is a disease rarely diagnosed in Poland (about 6 cases are reported per year), especially in comparison to Lyme borreliosis which is the most common tick-borne infection in Poland (about 3000 cases per year, with incidence- up to 10/100 000/year) [6,7]. Early Lyme borreliosis can present as meningitis with headache as a dominating symptom, however high fever is not so typical and tick-borne encephalitis (caused by flavivirus) should be considered as it was in the described case as well. The shift in the diagnosis was made when the general symptoms resolved but localised symptoms (the pain of the swollen lymph nodes) dominated. However, positive result of ELISA test for Lyme borreliosis which was available directly after admission influenced on the treatment decisions of doxycycline continuation with changed route of the drug administration. Penicillin and metronidazole were added as a non-specific treatment of the accompanying infection of the abdomen and pelvic region. Of the used antibiotics only doxycycline is among recommended treatment agents of tularaemia since most *F. tularensis* strains produce beta-lactamases [8]. No reports of tularaemia treatment with metronidazole are known to the authors.

Interestingly, oral doxycycline was started in the second week of the infection and was not effective in skin ulcer and did not prevented glandular form of tularaemia.

We can not exclude that resolution of the symptoms in the sixth week as it was described was an effect of both changing the route of administration of doxycycline and prolonged therapy with antibiotics (including metronidazole). Improvement in the sixth week of the disease might also reflect the natural course of infection with *F. tularensis*.

## Conclusion

In a case of prolonged, atypical skin reactions after a tick bite tularaemia should be considered.

Verification of ELISA test for Lyme borreliosis by western blotting test seems to be useful if diagnosis of accompanying infection is established.

## Abbreviations

CRP: C-reactive protein
ELISA: enzyme-linked immunosorbent test
hpf: high power field
